# Addressing key issues in HIV self-test program implementation for Black and Latino sexual minority men in the Southern United States: a multiphase study protocol

**DOI:** 10.1186/s43058-023-00395-6

**Published:** 2023-02-13

**Authors:** John Guigayoma, Sara J. Becker, Jason J. Ong, Mariano Kanamori, DeMarc Hickson, Lori M. Ward, Katie B. Biello, Tyler Wray

**Affiliations:** 1 Department of Behavioral and Social Sciences, Brown University School of Public Health, 121 S. Main St., Providence, RI 02912 USA; 2 Center for Dissemination and Implementation Science, Institute for Public Health and Medicine, Northwestern University, 633 N. Saint Clair St., Chicago, IL 60611 USA; 3 Faculty of Infectious and Tropical Diseases, London School of Hygiene & Tropical Medicine, Keppel St, London, WC1E 7HT UK; 4 Central Clinical School, Monash University, 99 Commercial Rd., Melbourne, VIC 3004 Australia; 5 Department of Public Health Sciences, University of Miami Miller School of Medicine, 1120 NW 10th St., FL 33136 Miami, USA; 6 Us Helping Us, People Into Living Inc., 3636 Georgia Ave. NW, Washington, DC 20010 USA; 7 University of Mississippi Medical Center, 2500 North State St., Jackson, MS 39216 USA; 8 Department of Epidemiology, Brown University School of Public Health, 121 S. Main St., Providence, RI 02912 USA; 9 The Fenway Institute, Fenway Health, 1340 Boylston St., Boston, MA 02215 USA; 10 Center for Health Promotion and Health Equity, Brown University School of Public Health, 121 S. Main St., Providence, RI 02912 USA

**Keywords:** HIV self-testing, Sexual minority men, Men who have sex with men, Consolidated Framework for Implementation Research, Discrete choice experiment, Willingness to pay, Linkage to care, HIV testing, HIV prevention

## Abstract

**Background:**

Black and Latino sexual minority men in the Southern United States have the highest HIV infection rates in the country. Increased HIV testing can help decrease onward HIV transmission through detecting previously undiagnosed infections. HIV self-testing is an evidence-based strategy to increase HIV testing among sexual minority men, but the implementation of this intervention in the Southern United States is limited. One implementation barrier is the lack of knowledge of Black and Latino sexual minority men’s preferences for various HIV self-testing program characteristics and their willingness to pay for these preferences. In addition, little is known about facilitators and barriers to initiating HIV self-testing programs from the perspectives of HIV prevention implementation decision-makers in this region.

**Methods:**

We will conduct an online discrete choice experiment among Black and Latino sexual minority men in the Southern United States (*n* = 300) to estimate this population’s preferences for the following HIV self-testing program characteristics: delivery strategy (home delivery, peer delivery, clinic pickup); delivery speed (same day, next day, 3 days, and 5 days); support (instructions only, during test, and 1 week after delivery); and price ($0, $20, $40, $50, $60). We will also use this choice data to generate willingness-to-pay estimates for each program characteristic. Guided by the Consolidated Framework for Implementation Research, we will then conduct semi-structured interviews (*n* = 30) with HIV prevention program decision-makers at various health organizations serving Black and Latino sexual minority men in the region to further understand facilitators and barriers to implementation of the most preferred HIV self-testing program design.

**Discussion:**

By gaining perspectives on HIV self-testing implementation from patients and providers, this project will build a roadmap for the initiation of HIV self-testing programs to decrease HIV incidence among one of the most disproportionately impacted populations in the USA.

Contributions to the literature

- Black and Latino sexual minority men in the Southern United States have the highest HIV infection rates in the country.
- One barrier to the implementation of evidence-based HIV prevention interventions, including HIV self-testing, is not knowing which program characteristics this population prefers.
- Conducting a discrete choice experiment to evaluate patient preferences for four key HIV self-testing program characteristics (delivery strategy, delivery speed, support, and price) will help practitioners design programs that better fit patients’ preferences.
- This study will provide guidance on how to translate patient preferences to implementation strategies using qualitative interviews guided by the Consolidated Framework for Implementation Research.

## Background

The Southern United States (South) has the highest rates of undiagnosed HIV infection in the nation [1], and Black and Latino sexual minority men experience the highest burden of HIV infections in this region [2]. Previous meta-analyses and a recent national randomized controlled trial in the USA show that directly supplying HIV self-tests to sexual minority men increases testing and detects previously unknown infections [3–5]. In the USA, the OraQuick In-Home HIV test (OraSure Technologies, Inc.) is the only rapid HIV self-test approved by the Food and Drug Administration [6] and consists of an oral swab antibody test that provides results in 20 min. Since research suggests that HIV transmission from undiagnosed sexual minority men to their sexual partners is a major driver of new HIV infections [7, 8], HIV self-testing with this device is one possible strategy to increase testing and decrease onward HIV transmission among Black and Latino sexual minority men. However, few healthcare organizations in the South offer HIV self-tests [9]. Research that addresses the barriers and facilitators to HIV self-test program implementation in the South can contribute to improved HIV testing for Black and Latino sexual minority men in this region.

The Consolidated Framework for Implementation Research (CFIR) describes several determinants that serve as barriers and facilitators to the adoption of evidence-based health interventions [10]. Formative qualitative research with key implementation stakeholders such as patients, program staff, medical providers, and administrators can identify which determinants are most critical to address when designing implementation strategies. Although HIV self-testing is one such evidence-based intervention, there is limited research from program stakeholders in the South on what healthcare settings need to implement it based on CFIR constructs. One notable exception is a study by King et al. of an HIV self-test peer distribution program within a mobile HIV pre-exposure prophylaxis clinic in Miami [11]. This study reported that program compatibility, organizational support, and feedback from clients facilitated a peer HIV self-test program, but costs to the organization, complexity, and external policies were barriers.

Research also suggests that understanding patient preferences for four aspects of HIV self-test programs, all of which pertain to the intervention characteristics domain of CFIR, may be especially critical to encouraging the use of this device among patients: how they get tests (delivery strategy), how quickly they get them (delivery speed), the support they get (support), and how much they pay (price) [12–14]. However, very few studies have examined these issues specifically among Black and Latino sexual minority men in the South [15]. In addition, resource restraints such as funding and staffing may pose challenges to HIV self-test program implementation, so knowing the tradeoffs patients are willing to make for their preferred programming can provide organizations with guidance on which program features to prioritize. Of these tradeoffs, patient willingness to pay is a particular concern because the associated costs of running an HIV self-test program are prohibitive for some organizations [12–14]. For this reason, knowing the extent to which Black and Latino sexual minority men in the South may engage in some cost sharing for their preferred services may serve as an additional facilitator to HIV self-test program implementation.

Understanding the optimal combination of HIV self-test program delivery strategy, delivery speed, support, and price among Black and Latino sexual minority men in the South can provide crucial information for the implementation of HIV self-test programs. Discrete choice experiments can serve as one survey method to examine this issue because they efficiently estimate patients’ preferences for multiple program attributes along with their willingness to pay for each attribute [16]. A discrete choice experiment can provide further insight into what Black and Latino sexual minority men in the South want from an HIV self-test program, which can contribute to increased implementation of this intervention for this underserved population.

### Specific aims

The overarching goal of this project is to identify the HIV self-test program characteristics that Black and Latino sexual minority men in the South most prefer and to understand the most important barriers and facilitators to implementing a program with these characteristics. Findings from this project will provide policymakers, organizational leaders, and HIV prevention staff with critical information to advance HIV prevention for Black and Latino sexual minority men in the region.

#### Aim 1 (phase 1)

To examine (a) the preferred HIV self-test delivery strategy, delivery speed, and support among Black and Latino sexual minority men in the South and (b) how much Black and Latino sexual minority men in the South are willing to pay for these program characteristics. To examine these issues, we will administer an online discrete choice experiment to 300 Black and Latino sexual minority men in the region.

#### Aim 2 (phase 2)

To understand the facilitators and barriers to implementation of the most preferred HIV self-test program from the perspectives of key implementation decision-makers in the South. To understand these factors, we will conduct qualitative interviews with 30 HIV prevention program decision-makers who serve Black and Latino sexual minority men in the region.

## Methods

### Study overview

Table 1 summarizes the timeline of this multiphase study protocol. Pilot testing for phase 1 (online discrete choice experiment with Black and Latino sexual minority men in the South) will take 3 months, followed by data collection over 9 months and data analysis over 6 months. Preliminary results from phase 1 will inform the potential HIV self-test program (delivery strategy, delivery speed, support, and price) we present to participants in data collection for phase 2 (qualitative interviews with HIV prevention program decision-makers in the South). Interview guide development for phase 2 will take 3 months, followed by data collection for 6 months and data analysis over 3 months. Phase 1 of this study received ethics approval from the Brown University Institutional Review Board. Phase 2 questions are dependent on phase 1 results, so we will obtain ethics approval for phase 2 after phase 1 is complete. We developed this protocol using the International Society for Pharmacoeconomics and Outcomes Research Good Research Practices for Conjoint Analysis Task Force checklist [17] for phase 1 and the Consolidated Criteria for Reporting Qualitative Research checklist [18] for phase 2.

**Table 1:** Study timeline for multiphase protocol on HIV self-test programming

	***Year 1***				***Year 2***			
	**Q1**	**Q2**	**Q3**	**Q4**	**Q1**	**Q2**	**Q3**	**Q4**
**Phase 1**								
Pilot testing	X							
Data collection		X	X	X				
Data analysis					X	X		
**Phase 2**								
Interview guide development					X			
Data collection						X	X	
Data analysis								X

#### Phase 1: Online discrete choice experiment with Black and Latino sexual minority men in the South

We will conduct an online discrete choice experiment to estimate preferences for HIV self-test program characteristics. Discrete choice experiments are based on random utility theory [19, 20], which states that preferences for a service are a function of the observable preferences of the characteristics of the program (attributes) along with some unobservable random error [21]. Discrete choice experiments gather preference data by presenting participants with a survey tool consisting of several “choice sets” of hypothetical program designs. These program designs are comprised of several attributes with varying characteristics (“levels”) for each attribute. Participants then choose their most preferred program design within each choice set [22]. The higher the preference for a program attribute level, the higher the probability participants would choose a program with that characteristic in the discrete choice experiment, and the greater the interest in a program with that characteristic in real-world settings [23]. Preference estimates from a discrete choice experiment can also be used to estimate willingness to pay for different levels of program attributes [24].

### Cognitive interviews

To aid in survey development, we will conduct pilot think-aloud cognitive interviews of the discrete choice experiment with 10 Black and Latino sexual minority men in the South (including 2 Spanish-speaking Black and Latino sexual minority men) (53). We will recruit these participants from a recruitment pool of eligible participants (see the “Recruitment” section in the “Phase 1: Online discrete choice experiment with Black and Latino sexual minority men in the South” section). This formative work will identify potential issues with the interpretation of discrete choice experiment instructions, prompts, and program attributes as well as feasibility based on the number of choice tasks. We will use these findings to refine the discrete choice experiment prior to data collection.

### Recruitment

We will recruit 300 Black and Latino sexual minority men from the South from a national online pool of sexual minority men interested in health studies. This pool was developed and successfully used across two current pragmatic, Web-based trials of sexual minority men who test for HIV infrequently (R01MH114891, PIs: Wray, Chan; P01AA019072, PIs: Wray, Monti) [25, 26]. Both trials use online marketing on popular social media (e.g., Facebook, Instagram) and gay-oriented dating websites/apps (e.g., Grindr, Jack’d) to recruit participants from various jurisdictions of the United States Department of Health and Human Services Ending the HIV Epidemic initiative in the South [27]. To recruit for the current protocol, we will sort through recruitment pool data to identify eligible participants not currently enrolled in either trial. We will reach out to potential participants via email and phone to provide basic information about this study.

Since current advertisements for the recruitment pool target primarily urban settings in the South to recruit a high volume of sexual minority men, we will supplement these recruitment efforts through additional online outreach focused on rural settings. Potential participants who click the advertisements will be directed to a unique screener specifically for this study. To allow for subgroup analyses based on race/ethnicity and rural/nonrural residence (see the “Analysis plan” section in the “Phase 1: Online discrete choice experiment with Black and Latino sexual minority men in the South” section), we will establish recruitment quotas (150 non-Hispanic/Latino Black and 150 Hispanic/Latino Black, 100 rural and 200 nonrural). We will periodically monitor these quotas and adjust recruitment strategies accordingly.

### Participants

Eligible participants will be (1a) assigned male sex at birth and currently identify as male or (1b) identify as a different sex than assigned at birth (transgender, including transwomen, transmen, and nonbinary individuals); (2) are at least 18 years old; (3) self-identify as Black or Latino; (4) have a primary residence in a state in the South; (5) have had anal or vaginal sex with man in the past year; (6) have never had an HIV-positive diagnosis; and (7) are fluent in English or Spanish.

### Procedures

Eligible individuals will provide informed consent to participate by signing an online form. To prevent fraud, we will verify participant identities and residence through LexisNexis, a commercially available online database of public records information which allows users to check personal information, such as names and addresses, against its database. Verified participants will complete all survey components in Qualtrics. Surveys will take participants approximately 30–45 min to complete. Participants will receive $25 for completing the survey. Spanish-speaking staff will translate all study materials into Spanish.

### Measures: discrete choice experiment

Participants will read a survey prompt before the discrete choice experiment that will provide introductory information on the OraQuick In-Home HIV Test, including administration procedures, diagnostic accuracy, and an image of the test. The prompt will also inform participants that the survey will assess their individual preferences of an HIV self-test program for routine HIV testing (every 6 months) and will maintain a neutral stance toward all possible choices (i.e., “no right answers”).

This discrete choice experiment will consist of four attributes: delivery strategy (home delivery, peer delivery, clinic pickup), delivery speed (same day, next day, 3 days, 5 days), support (instructions only, while you test, 1 week after delivery), and price ($0, $20, $40, $50, $60) (Table 2). The survey prompt will describe each attribute and attribute level (Table 3). We determined these attributes based on prior literature reviews and HIV self-test program designs currently implemented in the USA [28–31]. Delivery strategy levels are based on previous HIV self-test trials in the USA with the strongest evidence for increased HIV testing among sexual minority men [5, 26, 32, 33]. Delivery speed levels are based on the United States Postal Service and commercial delivery times [34, 35]. Support levels are based on strategies currently used by HIV self-test programs in the USA and previously researched strategies to provide HIV self-tests for peers [31, 36, 37]. Price levels are based on the range of possible HIV self-test prices in previous studies ($0–$40) [32, 38] as well as increased prices ($50, $60) for additional program characteristics Black and Latino sexual minority men may want, such as testing support or faster delivery times. Since HIV self-tests are currently available for $40 in retail settings with manufacturer instructions, we omitted possible programs that include higher HIV self-test prices for a similar service ($50 or $60 for 5-day home delivery with instructions only, $50 or $60 for same-day clinic pickup with instructions only). We also restrained delivery speed levels for clinic pickup to same-day only because longer delivery times are implausible in that setting.

**Table 2:** Attributes and attribute levels for the discrete choice experiment of HIV self-test program preferences

*Attribute*	*Attribute levels*
Delivery strategy	Home delivery, friend, clinic pickup
Delivery speed	Same day, next day, 3 days, 5 days
Support	Instructions only, while you test, after you test
Price	$0, $20, $40, $50, $60

**Table 3:** Attribute descriptions for the discrete choice experiment of HIV self-test program preferences

*Attributes*	*Prompt language*
**Delivery strategy**	
Home delivery	You order a test through a website of a popular local HIV testing clinic. You pay for the test online. The clinic mails a test to your home
Friend	You request a test from a close friend. Your friend brings you a test from a popular local HIV testing clinic. You pay your friend. Your friend gives that money to the clinic. The clinic never asks your name or contact info
Clinic pickup	You go to a popular local HIV testing clinic for a test. You pay the clinic for the test. You take the test home to use
**Delivery speed**	How long it takes to get a test when you request it
**Support**	
Instructions only	The test comes with written and picture instructions. It includes info for local HIV/STD testing. If you have a positive result, you can use this info to access medical care
While you test	A phone HIV test counselor (for home delivery or clinic pickup) or your friend (for friend delivery) guides you while you use the test. If you have a positive result, they can connect you to medical care
After you test	A phone HIV test counselor (for home delivery or clinic pickup) or your friend (for friend delivery) contacts you 1 week after you receive the test. If you have a positive result, they can connect you to medical care
**Price**	The cost to you of a test. The test accuracy is the same for all prices

Choice sets will consist of two hypothetical program designs (program A and program B) and a third “No HIV test” option. Table 4 presents an example choice set. We will include the no-test option to prevent potential bias from participants choosing an option when they otherwise would not [22]. Since previous randomized controlled trial research indicates high HIV self-test acceptability among sexual minority men in the USA [5, 26, 32], we do not anticipate the response rate for the no-test option to impede the calculation of preference estimates.

**Table 4:**
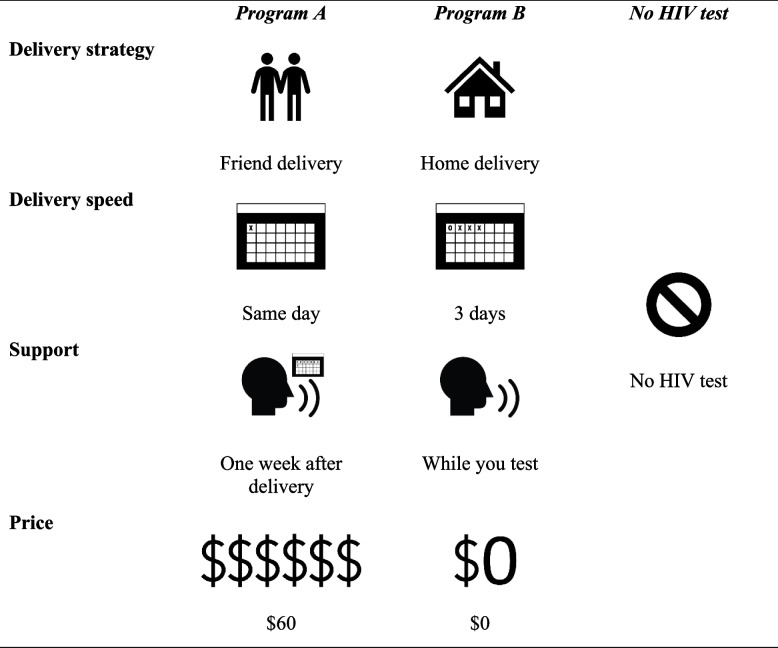
Example choice set for the discrete choice experiment of HIV self-test program preferences

The survey will randomize participants into one of 10 blocks. In line with other discrete choice experiments [39], each block will consist of eight choice sets (six for analysis followed by two for quality assurance) to achieve reliable outcome estimates while also preventing cognitive fatigue. The two quality assurance choice tasks will consist of (1) a repeat of the first choice task (consistency) and (2) a choice task in which one option is inherently superior to the other (rationality) (e.g., same-day delivery for $20 versus 5-day delivery for $60) [40]. This discrete choice experiment will use a d-efficient design to increase survey efficiency [41] by incorporating negative priors for price and delivery speed (D-error = 0.09). We used Ngene software (ChoiceMetrics) to design this discrete choice experiment.

### Measures: other factors

The online discrete choice experiment will also contain questions that will assess participant demographics, sexual risk, HIV testing history, and previous experience with HIV self-tests. The additional questions will also assess various cognitive factors for HIV testing such as HIV testing norms, attitudes, and self-efficacy, as these may be important factors that influence HIV self-test program attribute preferences and willingness to pay. Participants will also rate the general importance of several factors (e.g., convenience, privacy, social support) that guided their decisions in the discrete choice experiment. These measures are based on prior literature on constructs that may be especially important to Black and Latino sexual minority men in their preferences for HIV self-test programming [15, 42–44]. Response options for these factors will range from “Not important” (0) to “Very important” (4). We will also ask additional questions after the discrete choice experiment to gather information on the program selection process. These questions will include assessments of the importance of each attribute in participants’ decisions, ranging from “Not important” (0) to “Very important” (4), and a question on participants’ confidence in their responses, ranging from “Not confident at all” (0) to “Very confident” (4).

### Analysis plan

We will report participant characteristics using frequencies, means, ranges, and standard deviations. To understand preferences for program characteristics (aim 1a), we will use a mixed logit model, which accounts for individual-level clustering in preferences [21]. We will set the outcome of interest as the log odds of choosing program A over program B. The explanatory variables will consist of categorical variables representing delivery strategy (home delivery, peer delivery, and clinic pickup), delivery speed (same day, next day, 3 days, 5 days), and support (instructions only, while you test, 1 week after delivery) and a continuous variable representing price. The resulting model coefficients will be preference estimates for each attribute level and a separate coefficient for price. We will use effects coding to code the reference levels in each categorical variable as the negative sum of the other levels to generate non-zero estimates [21]. Higher preference estimates for an attribute level indicate higher relative interest in a program with that specific feature.

To understand the willingness to pay for the most preferred program characteristics (aim 1b), we will generate coefficients in units of dollars for each attribute level. This conversion consists of calculating negative ratios of the preference estimate for each attribute level to the preference estimate for price [24]. The resulting model will provide new coefficients that estimate how much individuals would pay for each HIV self-test program attribute option. For both models, we will also test for interaction effects based on key demographic categories such as age (less than 25 years old v. 25 years old or older), race/ethnicity (non-Hispanic/Latino Black v. Hispanic/Latino), and rural/nonrural residence. We will also test delivery strategy × price, delivery speed × price, and support × price interaction terms to understand if preference based on price differs between attribute levels (e.g., preference for home delivery from $0 to $60 versus preference for peer delivery from $0 to $60).

We follow Omre’s guidance for sample size calculations for discrete choice experiments, which recommends sampling enough participants to generate at least 500 observations for every level of each program attribute [45]. Based on the number of choice tasks (*N* = 6), the number of non-opt-out alternative options (*N* = 2, program A and program B), and the largest number of levels of any attribute (*N* = 5, the five levels in the price attribute), our study needs approximately 209 participants to analyze main effects. Based on our target of 300 participants, we expect to recruit enough individuals to generate robust preference estimates. We will also report on validity checks for discrete choice experiment responses, including proportions of participant nonresponse and failure of the quality assurance tests. If there are substantial validity issues based on the quality assurance tests, we will report results with and without these responses for comparison. We will conduct all analyses using NLOGIT software (Econometric Software, Inc).

By the end of this phase, we will have identified the relative preferences for HIV self-test delivery strategies and support strategies among this sample of Black and Latino sexual minority men in the South as well as their willingness to pay for each. We will use the most preferred HIV self-test program characteristics from phase 1 to inform qualitative research with HIV program decision-makers in phase 2.

#### Phase 2: Qualitative interviews with HIV prevention decision-makers in the South

In phase 2, we will explore determinants of HIV self-test program implementation using the preferred program attributes and corresponding willingness-to-pay estimates identified in phase 1 among a sample of HIV prevention decision-makers in the South. We will recruit *n* = 30 HIV prevention program decision-makers (e.g., health department directors, community-based organization leaders) for in-depth qualitative interviews focused on a thorough understanding of determinants (i.e., facilitators and barriers) of HIV self-test program implementation based on the CFIR model.

### Interview guide development

We will develop a semi-structured interview guide to understand determinants of implementation of the preferred HIV self-test program identified in phase 1. We will use the publicly available CFIR Universal Interview Guide, which has been widely used across a variety of populations and provides example qualitative interview questions based on CFIR determinants (Table 5) [46]. Based on previous HIV self-test program research, this interview guide will consist of constructs across all five CFIR domains: (1) intervention characteristics (e.g., relative advantage of the HIV self-test programs compared to other program designs), (2) stakeholder characteristics (e.g., attitudes toward the HIV self-test programs), (3) inner setting (e.g., compatibility of the HIV self-test programs with current medical services), (4) outer setting (e.g., policies and incentives to implement the HIV self-test programs), and (5) process (e.g., strategies to support HIV self-test program implementation) [10].

**Table 5:** Example HIV self-test program qualitative interview questions using the Consolidated Framework for Implementation Research (CFIR)

*CFIR domain*	*Questions*	*Probes*
Intervention characteristics	How does this HIV self-test program compare to other similar existing HIV testing programs in your setting?	What advantages does this HIV self-test program have compared to existing programs? What disadvantages does this HIV self-test program have compared to existing programs?
Outer setting	What kind of local, state, or national performance measures, policies, regulations, or guidelines would influence your decision to implement this HIV self-test program?	How would this HIV self-test program affect your organization’s ability to meet these measures, policies, regulations, or guidelines?
Inner setting	How would current HIV testing programming at your organization affect the implementation of this HIV self-test program?	How would current HIV testing programming facilitate the implementation of this HIV self-test program? How would current HIV testing programming hinder the implementation of this HIV self-test program?
Individual characteristics	What do you know about the HIV self-test or its implementation? Do you think this HIV self-test program will be effective in your setting? Why or why not?	Do you have any feelings of anticipation about implementing this HIV self-test program? Stress? Enthusiasm? Why?
Process	Can you describe a plan for implementing this HIV self-test program in your setting?	How detailed is the plan? Who knows about it? What is your role in the planning process? Who else is involved in the planning process? What are their roles?

### Recruitment

We will recruit health department and community-based organization directors in the South (*n* = 30) who have decision-making authority over HIV testing programs.

We will purposively sample to include variation in perspectives based on organization type (community-based organization, hospital/medical center, health department), state, and type of HIV testing offered (rapid HIV testing v. laboratory-based blood testing). We will recruit participants through direct referral from three team members based in the South with extensive connections to HIV testing facilities across the region. We will also recruit through Internet searches, listservs, and participant referrals. We will report the number of potential participants we approached and the number who refused to participate.

### Participants

Participants must currently (1) be at least 18 years old; (2) have decision-making authority over HIV testing programs at a regional or state health department, a community-based organization providing HIV testing services, or an HIV/sexually transmitted infection testing clinic in the South; (3) have served in this role at their current organization for at least 1 year; and (4) have access to a computer with Internet access and Zoom videoconferencing. We will ask interested participants to verbally verify these criteria before participating.

### Procedures

We will ask participants to schedule a time to conduct a Web-based video interview via Zoom. Participants will provide informed consent through an online form. A cisgender male doctoral candidate based in the Northeastern United States with graduate-level training in qualitative research and previous experience implementing HIV testing programs will conduct the interviews using a semi-structured interview guide. The interviewer will have no prior relationship to the participants or their organizations. At the beginning of the interview, the interviewer will state that the goal of the interview is to identify barriers and facilitators to an HIV self-test program for Black and Latino sexual minority men in the South based on prior survey research. Interviews will last about 1 h. We will record interviews via Zoom and will use Zoom’s automated transcription services to generate interview transcripts. At the end of each interview, participants will complete a brief survey to provide basic data on HIV prevention work history, organizational characteristics, and personal demographics. Within 1 week of each interview, the interviewer will review all audio transcripts for accuracy and write corresponding field notes indicating contextual details and nonverbal cues. We will not return transcripts to participants for review. Participants will receive $100 for participating in the qualitative interview and to cover work time spent.

### Qualitative interview analysis

Two independent qualitative analysts will conduct thematic analysis under the guidance of a third analyst, a clinical psychologist with expertise in qualitative methods for implementation science. Thematic analysis is a deductive approach that allows researchers to identify which selected CFIR constructs are important to program decision-makers and how these constructs influence HIV self-test program implementation [47]. After data cleaning, the analysts will develop a set of codes based on meaningful recurring concepts and will assign these codes to data segments accordingly. From these coded data, the analysts will identify larger themes and corresponding subthemes that they will organize within a “thematic map” that will show potential thematic relationships. The three analysts will then review the validity of this thematic map through recurring reviews of interview transcripts and refine it accordingly. Once we have established that the thematic map is internally consistent and sufficiently describes the data, we will report this map of major and minor themes with corresponding participant quotations that depict determinants of HIV self-test program implementation. We will use NVIVO software (QSR International) to manage these data.

The two primary analysts will both code the first two interviews to achieve interrater reliability. One analyst will code subsequent interviews, and the second will code every fifth interview to maintain integrity. The analysts will meet weekly and regularly consult with team members with expertise in implementation science and HIV testing to identify themes and construct and refine the thematic map. We will send a 1-page visual summary of the findings to participants and provide the opportunity for participant feedback. Following the finalization of the thematic map, we will enter the identified CFIR determinants into the CFIR-Expert Recommendations for Implementation matching tool. We anticipate that this tool will generate a set of specific implementation strategies that can serve as potential candidates to implement the HIV self-test program identified in phase 1 for Black and Latino sexual minority men in the South, which will lay the groundwork for future work with our team [48].

## Discussion

This multiphase research aims first to identify the ideal attributes of an HIV self-test program, then to identify determinants of implementation of a program with these attributes, and finally to generate a set of potential implementation strategies to address these determinants. Since Black and Latino sexual minority men in the South have the highest HIV infection rates in the country, the implementation of HIV self-test programming can potentially contribute to increasing HIV testing and a corresponding decrease in onward HIV transmission. The implementation strategies identified in this work will form the basis for a future hybrid 1 effectiveness-implementation trial [48]. The primary objective of this trial will be to test the effectiveness of the HIV self-test intervention identified in phase 1, and the secondary objective will be to simultaneously examine the implementation potential of HIV self-test programming using some of the implementation strategies identified in phase 2.

This research is subject to several limitations. First, since the discrete choice experiment in phase 1 is a stated preference exercise, BLMSM may overestimate their self-assessed preference for HIV self-test delivery options and willingness to pay for these options. Although this limitation may lead to inflated estimates, previous research has shown that discrete choice experiments have reasonable external validity for real-world program design [23] and have stronger validity for assessing willingness to pay than other survey methods [49]. In addition, the qualitative interviews in phase 2 are subject to social desirability bias, either in terms of perceived reactions to the participants’ responses by their workplaces or by the interviewer. To address this issue, interview procedures will emphasize interviewer neutrality and participant confidentiality.

Despite these limitations, this research will be the only study to date to estimate preferences for HIV self-test delivery strategy, delivery speed, support, and corresponding willingness to pay in a sample of Black and Latino sexual minority men in the South. Knowledge of patient preferences is vital to the design of scalable HIV self-test programs that can be implemented and sustained in this underserved region. Finally, this study provides guidance on the application of discrete choice experiment results to HIV prevention programming through implementation science methods. This multiphase protocol provides researchers with an example methodological approach to translate preference data from patients to the design of real-world intervention components and implementation strategies to decrease HIV transmission.

## Data Availability

Data collection is currently in progress, so data is only currently available to the research team. All study data will be available upon request from the corresponding author upon completion of data collection. We will not make this data publicly available because it contains information that could compromise research participant consent.

## Abbreviation

CFIR: Consolidated Framework for Implementation Research
